# Regional risk of *tuberculosis* and viral hepatitis with tumor necrosis factor-alpha inhibitor treatment: A systematic review

**DOI:** 10.3389/fphar.2023.1046306

**Published:** 2023-01-20

**Authors:** Nina Jahnich, Peter D. Arkwright

**Affiliations:** Lydia Becker Institute of Immunology and Inflammation, Manchester Incubator Building, University of Manchester, Manchester, United Kingdom

**Keywords:** biologic, TNFa blockade agents, tuberculosis, epidemiology, viral hepatitis, inflammatory disease, guidelines and recommendations, autoimmune disease

## Abstract

**Background:** TNFα inhibitors are regularly used to treat autoimmune diseases. Tuberculosis (TB) and viral hepatitis B are considered potential infectious complications, and screening and surveillance are therefore recommended. Current guidelines do not take into account regional differences in endemicity of these infections.

**Methods:** A systematic literature review of TB and viral hepatitis in patients receiving TNFα-inhibitors was performed, searching in PubMed, Embase, MEDLINE and Web of Science databases. Studies were selected against predefined eligibility criteria and assessed using the Newcastle-Ottawa scale. The number of TB and viral hepatitis cases/1,000 TNFα-inhibitor patients were evaluated, and regional variation compared.

**Results:** 105 observational studies involving over 140,000 patients were included. Overall, 1% of patients developed TB or viral hepatitis B. TB cases/1,000 TNFα-inhibitor patients were 4-fold higher in Asia, Africa, and South America than in Europe, North America, and Australasia where only 0%–0.4% of patients developed TB. Hepatitis B cases/1,000 patients were over 15-fold higher in countries with high prevalence (China, Taiwan, South Korea, Thailand) compared with low prevalence (*p* < 0.00001) where only 0.4% of patients developed hepatitis B. Only three of 143 patients developed viral hepatitis C, and there was insufficient data to allow regional sub-analysis.

**Conclusion:** TB and viral hepatitis B infections in patients treated with TNFα inhibitors are largely confined to countries with high prevalence of these infections. As only 1/2,500 patients in low prevalence countries treated with TNFα inhibitors develop TB or viral hepatitis B, we suggest an individualized, risk-based approach, rather than universal screening for all patients.

## Introduction

Dysregulated tumor necrosis factor alpha (TNFα) signaling plays a central role in the pathogenesis of many inflammatory diseases such as rheumatoid arthritis (RA), inflammatory bowel disease (IBD) and psoriasis (PsO) (Ettehadi et al., 1994; Olsen et al., 2007; Sands and Kaplan, 2007; Tracey et al., 2008). Anti-TNFα biologics improve patient outcome, particularly in those who do not respond to conventional immune suppressants (van der Heijde et al., 2005; Colombel et al., 2007). There are currently five approved TNFα-inhibitor therapies: infliximab, adalimumab, golimumab, and certolizumab pegol are monoclonal-antibody therapies, whilst etanercept is a receptor fusion protein.

Tuberculosis (TB), hepatitis B (HBV) and hepatitis C virus (HCV) are considered potential infectious complications of TNFα-inhibitor therapies (Salvana and Salata, 2009). Concerns regarding TB risk followed a 2001 study, in which 70 TB cases (0.03%) were reported in 249,131 patients receiving infliximab or etanercept, 62 from the US and low-risk countries in Europe (Keane et al., 2001). Fifty-six percent had extrapulmonary TB and one-quarter had disseminated TB. Fifty-five patients had received another immunosuppressant. Subsequent clinical-trial, post-marketing data and registry case series have reported further cases of TB (Fischkoff, 2001; Mohan et al., 2004; Wallis et al., 2004). It is now widely accepted that TNFα inhibitors are associated with an increased risk of TB, although a definite causal relationship has not been established.

Because of evidence of HBV and HCV reactivation in patients receiving immunosuppressive therapies such as glucocorticoids and chemotherapy (Vento et al., 2002), concerns have also been raised regarding a risk of viral hepatitis with TNFα-inhibitor use. There have been reported cases of HBV and HCV reactivation in TNFα-inhibitor patients in clinical practice, although attribution of these infections to TNFα inhibitors is circumstantial (Michel et al., 2003; Pérez-Alvarez et al., 2011).

The implication is that expert opinion and clinical guidelines recommend that all patients receiving TNFα inhibitors undergo screening and monitoring for TB and viral hepatitis infections. However, current screening guidelines do not consider regional differences in the endemicity of these infections. Two-thirds of the 10 million new TB infections reported globally in 2020 were from only eight countries in Asia and Africa (World Health Organization (WHO), 2021). China and India reported some of the highest numbers of new TB cases in 2020, an estimated 59 and 188 cases/100,000 of the population respectively, compared to an estimated 2.4 TB cases/100,000 of the population in the United States. Western European nations, Canada, Australia, and New Zealand also reported a low incidence of TB (Pai et al., 2016). China (≥8%), South-East Asian and African regions report the highest prevalence of chronic HBV infection, whilst Europe, Western Asia, and North America report a low prevalence of <1% (World Health Organization (WHO), 2017; European Association for the Study of the Liver (EASL), 2018). China, Pakistan, India, Egypt, and Russia account for half of worldwide HCV infections, whilst only a small percentage occur in Western countries (Manns et al., 2017).

Previous systematic reviews have reported an increased risk of TB in patients receiving TNFα inhibitors (Ai et al., 2015; Zhang et al., 2017), with a greater frequency of infection in Asia and South America compared with Western Europe and North America (Sartori et al., 2020). However, these systematic reviews were largely limited to randomized clinical trials, restricted to patients with rheumatic diseases and lacked real-world data. Previous systematic reviews and meta-analyses estimate a low HBV reactivation rate (Cantini et al., 2014; Moghoofei et al., 2018), are limited to patients with rheumatic diseases and are outdated.

No previous systematic review has investigated interregional differences in TB infection in TNFα-inhibitor patients across all inflammatory diseases. There are no reports assessing the interregional differences in both HBV and HCV infection risk in patients treated with TNFα-inhibitor therapies. As the number of TNFα inhibitor prescriptions increase worldwide, there is an urgent need to understand regional-specific risks of TB and viral hepatitis infection, allowing for an evidence base for infection screening and surveillance. The aim of this systematic review was therefore to assess the interregional differences in TB and viral hepatitis in patients exposed to TNFα inhibitors using real-world data. The study compared the frequency of these infections in TNFα-inhibitor patients across continental subgroups, as well as subgroups according to high or low infection prevalence in the general population.

## Materials and methods

### Protocol and registration

This systematic review was conducted in accordance with the Preferred Reporting Items for Systematic Reviews and Meta-Analyses (PRISMA) guidelines (Page et al., 2021). The review protocol was registered at PROSPERO (registration number: CRD42022358834).

### Search strategy

A systematic search was performed for published observational studies, including post-marketing reports, that reported the number of cases of TB, HBV and/or HCV among patients exposed to any of the five approved TNFα inhibitors. A systematic literature search of the PubMed, Embase, MEDLINE and Web of Science databases was conducted up to 23rd May 2022, using predetermined key search terms and combinations thereof (Supplementary Appendix SA). The database search was supplemented by screening recent existing systematic reviews related to the topic for additional relevant studies not identified in the literature search (Cantini et al., 2014; Zhang et al., 2017; Sartori et al., 2020).

### Eligibility criteria

Studies were included according to predefined inclusion criteria:

#### Participants

Patients with any rheumatic, dermatologic or inflammatory disease exposed to any of the five approved TNFα inhibitors. No limitation on age, length of time receiving treatment or length of follow-up.

#### Interventions

Exposure to at least one TNFα inhibitor (infliximab, adalimumab, etanercept, golimumab, certolizumab pegol), with or without concomitant standard treatment.

#### Comparators

Patients not exposed to TNFα inhibitors.

#### Outcomes

Number of cases of TB, HBV, and/or HCV infection or reactivation. Geographic location of study.

#### Study design

Published observational studies, post-marking reports. No limitation on location of study or date of publication.

Non-English language publications, letters, case reports, clinical trials, meta-analyses, and abstracts were excluded. Publications which did not state the location of the study, or did not report number of cases of TB, HBV and/or HCV were also excluded.

### Study selection

Screening was performed by NJ and then output reviewed by PDA. Studies retrieved by the search strategy were exported to EndNote 20 and the total number of records was documented. Duplicated studies were removed using the EndNote ‘Find Duplicates’ automated tool, as well as manually by screening of publication title. Remaining records were then screened by title and abstract to produce a list of publications for full-text review, excluding unrelated and irrelevant articles. Any publications which could not be confidently excluded by title and abstract underwent full-text review to assess eligibility, according to the inclusion and exclusion criteria. Following full-text assessment for eligibility, a final list of publications for inclusion in the systematic review was produced.

### Data extraction and quality assessment

NJ performed data extraction and risk of bias evaluation. For each included study, the following primary outcomes were extracted: number of patients exposed to TNFα inhibitors, number of patients who developed active TB, HBV and/or HCV (diagnosed through any screening method recommended in the guidelines), and geographical study location. As well as essential publication details (date, authors, type of study), the secondary outcomes extracted were: TNFα inhibitor(s) used, disease(s) treated, use of concomitant treatment and prophylaxis, serological infection status prior to TNFα-inhibitor therapy, time elapsed from exposure to infection, management of infection and number of infection-related deaths.

The quality of the studies was assessed for bias using the Newcastle-Ottawa quality assessment scale for cohort studies (Wells et al., 2000). According to the Newcastle-Ottawa scale, a maximum score of 9 can be awarded to a study, based on customizable categories relating to the selection of the study groups, comparability of the groups, and ascertainment of outcome. In this systematic review, studies with a score ≥5 were considered to have low risk of bias. Full details of the customizations made to the Newcastle-Ottawa scale are detailed in the Supplementary Appendix SB.

### Statistical analysis

Included studies were divided according to the infectious complication they assessed. For each infectious complication, studies were further organized by geographic location for subgroup analyses. Studies were organized into subgroups by continent, as well as into subgroups of high or low burden of the infection in the population of the study country. The primary endpoint for each subgroup was evaluated and expressed as number of TB, HBV, or HCV cases/1,000 patients exposed to TNFα inhibitors. The number of cases/1,000 patients were compared between subgroups. Statistical comparisons between subgroups were performed using Chi-squared test, with a 0.05 significance level (Stangroom, 2022).

## Results

**FIGURE 1:**
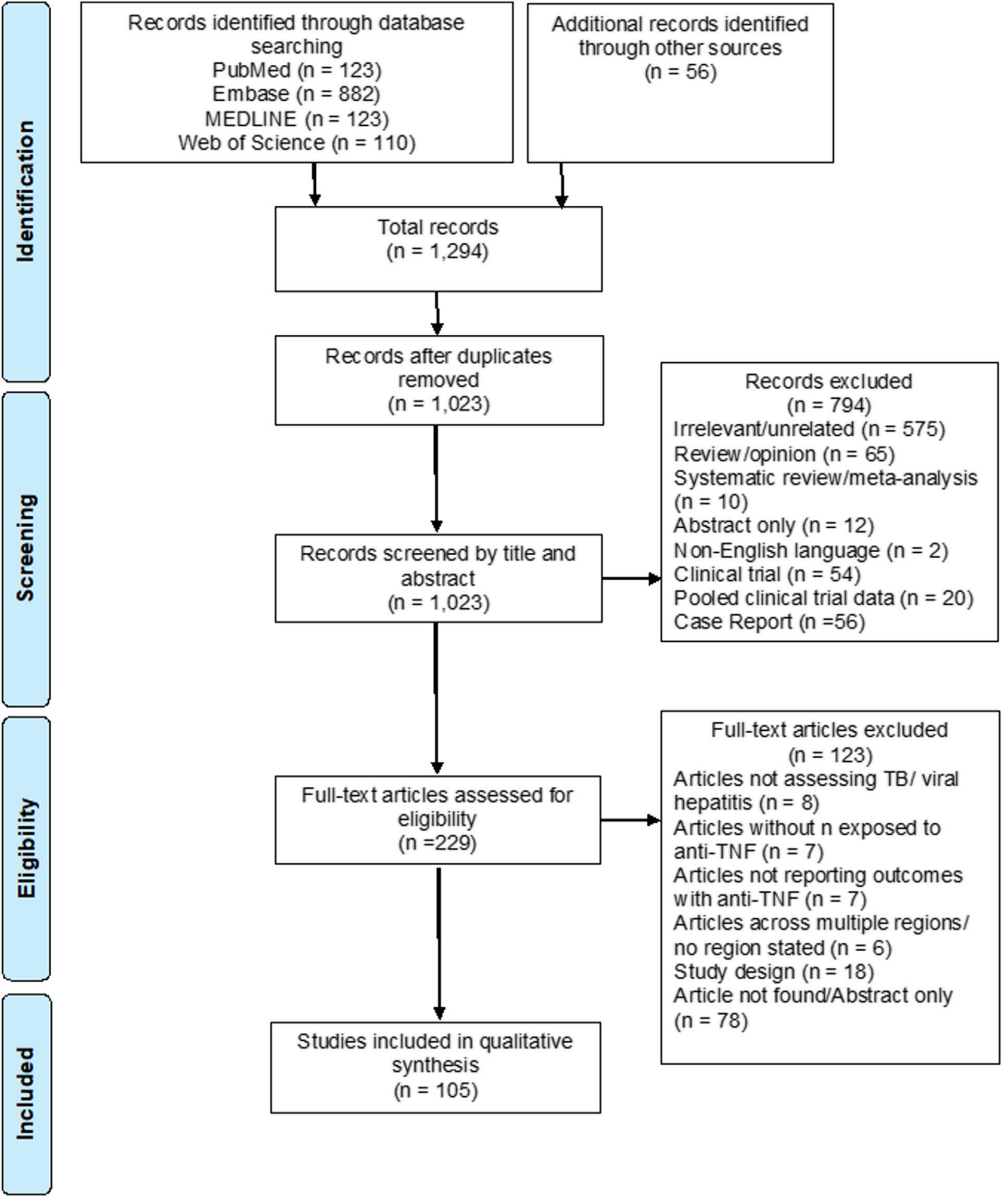
PRISMA flow diagram of study identification, screening, and selection for inclusion in this systematic review.

The results of the study selection strategy, including reasons for exclusion, are outlined in the PRISMA flow diagram (Figure 1). 1,238 records were identified through the database search: 123 records in PubMed, 882 in Embase, 123 in MEDLINE and 110 in Web of Science. Fifty-six additional publications were identified through screening the references of recent relevant systematic reviews, giving a total of 1,294 records. After removal of duplicates, 1,023 records remained and were examined by title and abstract. 794 articles were excluded as they did not meet the inclusion criteria. The remaining 229 articles were reviewed in full text for eligibility.

A total of 105 articles ultimately met the inclusion criteria and were included in the systematic review, with publication years ranging from 2004–2022. Eighty-four included studies reported the number of cases of TB infection or reactivation with TNFα inhibitors, 23 studies reported the number of cases of HBV infection or reactivation, and six studies reported the number of cases of HCV reactivation. Eight of these studies investigated more than one infectious complication and were included in more than one group.

### Assessment of risk of bias

The risk of bias of the 105 included studies was evaluated according to categories outlined in the Newcastle-Ottawa scale (Wells et al., 2000) (Supplementary Appendix SB). Studies with a score of <5 out of a maximum of nine were considered to have potential risk of bias. A score of <5 was awarded to only four studies. All four studies investigated TB infection with TNFα inhibitors.

### TB risk with TNFα inhibitors

Eighty-four observational studies published from 2004–2022 were identified, in which the number of cases of TB infection or reactivation was reported in patients receiving TNFα inhibitors. Of these observational studies, four were post-marketing reports. Eighty of the 84 studies reported only TB infections, whist the remaining four reported both TB and HBV infections. The studies investigated patients being treated for a broad range of inflammatory conditions, most commonly RA, ankylosing spondylitis (AS), psoriatic arthritis (PsA), PsO, ulcerative colitis (UC), and Crohn’s disease (CD). Included studies investigated both children and adults. Extended study characteristics are outlined in Supplementary Appendix Table SC1.

The median number of study participants was 8,359 (range 25–16,742). A total of 138,592 patients were exposed to at least one TNFα inhibitor. Of those exposed to TNFα inhibitors, 1,320 developed active TB (1% of patients). Ten studies reported no cases of TB development, whilst 12% of TNFα-inhibitor exposed patients developed active TB in an Indian cohort (Agarwal et al., 2018). Overall, there were 10 TB cases/1,000 TNFα-inhibitor-exposed patients.

When considering only studies with a low risk of bias, 137,059 patients were exposed to TNFα inhibitors, of whom 1,313 developed active TB (1% of patients). Frequency of TB was 10 cases/1,000 exposed patients in studies with a low risk of bias. There was no significant difference in the number of TB cases/1,000 patients between low risk of bias studies and all studies included (*p* = 0.9), and thus the quality of the included studies was deemed sufficient.

### Subgroup analysis of TB by continent

Studies which reported the number of cases of TB in TNFα-inhibitor patients were subdivided according to continental location for subgroup analysis. Of the 84 included studies, 52 were conducted in Asia, 17 in Europe, seven in North America, five in South America, two in Africa and one in Australasia (Table 1).

**TABLE 1:** Interregional differences in the frequency of TB infection in patients receiving TNFα inhibitors.

Geographic region	# Studies	Total patients	Infected patients	% Infected patients	TB cases/1,000 patients	*p*-value
World	84	138,592	1,320	1.0	10	<.00001
Asia	52	79,428	1,077	1.4	14	
Europe	17	38,719	171	.4	4	
N. America	7	18,685	22	.1	1	
S. America	5	874	30	3.4	34	
Africa	2	260	20	7.7	77	
Australasia	1	626	0	.0	0	
High-burden continents	59	80,562	1,127	1.4	14	
Low-burden continents	25	58,030	193	.3	3	<.00001
High-burden countries	28	33,021	626	2.0	20	
Remaining countries	56	105,571	644	.6	6	

Statistical comparison was performed using the Chi-squared test, with a 0.05 significance level. The number of TB cases/1,000 patients was compared between distinct subgroups: studies in continents with a high TB burden (Asia, South America, Africa) compared to continents with a low TB burden (Europe, North America, Australasia); and studies undertaken in the top 30 “high-burden countries” for TB (Brazil, China, India, South Africa, and Thailand) versus the remainder. Taiwan and Hong Kong were considered as part of China in this analysis.

**FIGURE 2:**
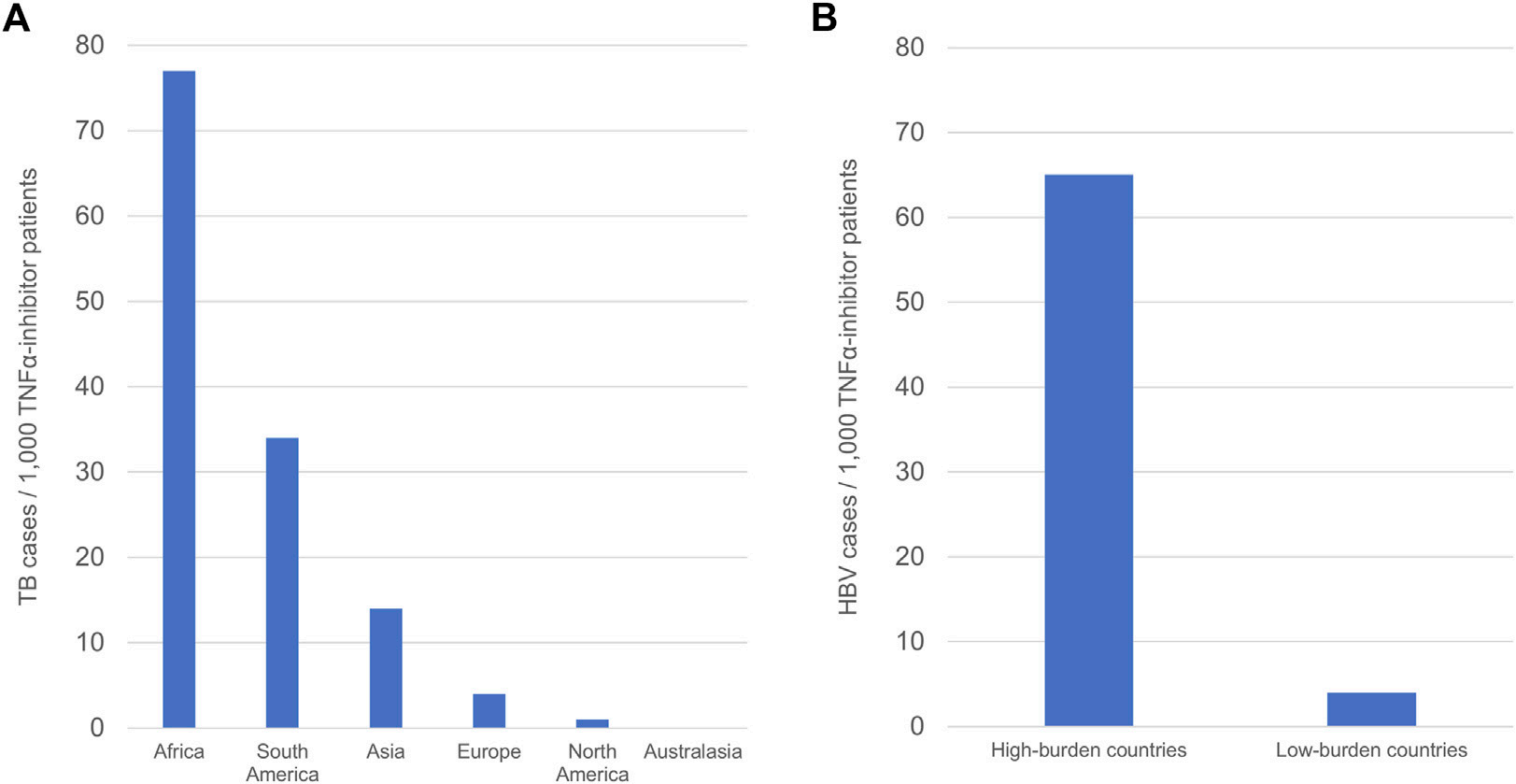
Regional differences in the frequency of TB and HBV infection in TNFα-inhibitor patients. **(A)** Continental differences in TB frequency in TNFα-inhibitor patients. **(B)** Differences in HBV frequency according to regional burden. Frequency is defined as number of cases/1000 TNFα-inhibitor patients.

A total of 79,428 patients were exposed to at least one TNFα inhibitor in ten countries in Asia, with 1,077 cases of active TB reported (1.4% of patients). This corresponds to 14 cases/1,000 exposed patients. Two of 52 Asian studies reported no cases of active TB (Torii et al., 2016; Al-Sohaim et al., 2021). 38,719 patients received TNFα inhibitors in ten countries in Europe, in whom 171 cases of active TB were reported (0.4% of patients), corresponding to 4 cases/1,000 exposed patients. Three of 17 European studies reported no cases of active TB (Bracaglia et al., 2012; Atteno et al., 2014; Tarkiainen et al., 2015). In North America (United States and Canada), 18,685 patients were exposed to at least one TNFα inhibitor. Twenty-two cases of active TB were reported in this population (0.1% of patients), with 1 case/1,000 exposed patients. Three of seven North American studies reported no cases of active TB (Aggarwal et al., 2009; Rahman et al., 2016; Rahman et al., 2020a). Of the five studies conducted in South America, were all located in Brazil. 874 patients received TNFα inhibitors, and 30 cases of active TB developed in this population (3.4% of patients). This corresponds to 34 cases/1,000 exposed patients. Both African studies took place in South Africa. 260 patients received at least one TNFα inhibitor. Twenty cases of active TB were reported in a single study (du Toit et al., 2020), with no cases of active TB in the other study (Pettipher et al., 2016) (7.7% of patients). Overall, there were 77 TB cases/1,000 exposed patients across African studies. A single study was conducted in Australasia, across New Zealand and Australia (Lawrance et al., 2010). In this study 626 patients were exposed to TNFα inhibitors, with no cases of active TB. The greatest number of TB cases/1,000 patients was observed in Africa, followed by South America and Asia, whilst the lowest numbers of TB cases/1,000 patients were observed in Australasia, North America, and Europe (Figure 2).

When considering continental differences in the development of active TB in TNFα-inhibitor patients, Asian, South American, and African studies comprised the subgroup of studies performed in continents with high TB burden, whilst European, North American, and Australasian studies comprised the subgroup of studies performed in continents with low TB burden. TB cases in the high-burden continents demonstrated a combined frequency of 14 TB cases/1,000 patients, whilst combined frequency in the low-burden continents was 3 TB cases/1,000 patients (Table 1). These data show a statistically significant difference in frequency of TB between studies in Asia, South America and Africa compared to Europe, North America, and Australasia (*p* < .00001).

### Subgroup analysis of TB by regional burden

To account for the fact that general prevalence of TB may vary between countries within the same continent, a subgroup comparison according to regional burden was performed. The World Health Organization (WHO) has established a list of 30 ‘high-burden countries’ for TB [World Health Organization (WHO), 2021]. These are the top 20 countries with the highest absolute number of new TB cases in 2019, and the top ten countries with the highest number of new cases/100,000 of the population in 2019 not already included in the list, that exceed the threshold of 10,000 new cases/year. When considering the top 30 high-burden TB countries, 28 of the included studies took place in these regions, including studies in Brazil, China (including Taiwan and Hong Kong), India, South Africa, and Thailand (Table 1). There were 20 TB cases/1,000 exposed patients in those exposed to TNFα inhibitors in these studies, compared to 6 cases/1,000 exposed patients in the remaining studies (Table 1). There was a statistically significant difference in the number of TB cases/1,000 patients between studies performed in the top 30 high-burden TB countries and those performed outside of these countries (*p* < .00001). Adding South Korea as a high TB burden country, 42 studies took place in high-burden regions with 19 cases TB/1,000, compared to 4 cases TB/1,000 in low-burden countries (*p* < .00001).

### HBV risk with TNFα inhibitors

Twenty-three observational studies published from 2009 to 2022 were identified, which reported the number of cases of HBV infection or reactivation in patients receiving TNFα inhibitors. One of these studies was a post-marketing report. Four studies reported both HBV and HCV infections, and four studies reported both HBV and TB infections. Extended study characteristics are outlined in Supplementary Appendix Table SC2.

The median number of study participants was 845 (range 4–1,693). A total of 3,554 patients received at least one of the five approved TNFα inhibitors. Patients were treated across a broad spectrum of rheumatological, dermatological and inflammatory bowel diseases, most commonly RA, AS, PsA, PsO, UC, and CD. Forty-seven cases of active HBV were reported (1.3% of patients), defined by the presence of previously undetectable HBV-DNA, HBV-DNA viral load increase, or the appearance of the HBV surface antigen, HBsAg, in serum. Eight studies reported zero cases of active HBV, with a high of 43% of TNFα-inhibitor-exposed patients experiencing HBV reactivation in a Taiwanese study (Cho et al., 2012). Overall, 13 HBV cases/1,000 exposed patients occurred in the included studies.

### Subgroup analysis of HBV by continent

Studies were subdivided by continental location (Table 2). A total of 14 studies were conducted in Asia and nine studies were conducted in Europe. No studies were conducted in Africa, Australasia, or the Americas.

**TABLE 2:** Interregional differences in the frequency of HBV infection in patients receiving TNFα inhibitors.

Geographic location	# Studies	Total patients	Infected patients	% Infected patients	HBV cases/1,000 patients	*p*-value
World	23	3,554	47	1.3	13	
Asia	14	3,143	43	1.4	14	.5
Europe	9	411	4	1.0	10	
High-burden regions	6	339	22	6.5	65	<.00001
Low-burden regions	16	3,024	11	.4	4	

Statistical comparison was performed using the Chi-squared test, with a 0.05 significance level. The number of HBV cases/1,000 patients was compared between distinct subgroups: studies in Asia vs. studies in Europe, and studies in countries with a high (≥8%) chronic HBV burden (Taiwan, South Korea, Thailand, China) vs. studies in countries with a low (<8%) chronic HBV burden (Italy, France, Spain, Greece, Turkey, Japan). In the latter comparison, a study across multiple Asian countries was not included (Lee et al., 2022), and hence the sum of studies and patients in this comparison does not equal that of all studies.

3,143 patients were exposed to TNFα inhibitors in six countries in Asia. Forty-three cases of HBV infection or reactivation were reported in this population (1.4% of exposed patients). Two of 14 studies in Asia reported no cases of active HBV (Sayar et al., 2020; Namba et al., 2022). Frequency of HBV across Asian studies was 14 cases/1,000 patients. 411 patients received TNFα inhibitors in four countries in Europe, with four cases of HBV reported across three studies (1% of exposed patients) (Garcia-Vidal et al., 2009; Vassilopoulos et al., 2010; Morisco et al., 2013). The remaining six studies reported no cases of active HBV. Frequency of HBV across European studies was 10 cases/1,000 patients. The number of HBV cases/1,000 exposed patients in studies performed in Europe was not statistically significant in relation to studies performed in Asia (*p* = .5).

### Subgroup analysis of HBV by regional burden

Studies were also subdivided into those performed in countries with high HBV burden, defined as ≥8% prevalence of chronic HBV, and those performed in low burden countries with <8% prevalence of chronic HBV (Yuen et al., 2018) (Table 2). High-burden countries included Taiwan, South Korea, Thailand, and China, whilst low-burden countries included Italy, France, Spain, Greece, Turkey, and Japan. A single study conducted across multiple Asian nations of differing HBV burden was excluded from this analysis (Lee et al., 2022). Three-hundred and thirty-nine patients were exposed to TNFα inhibitors in high-burden countries, across six studies. Twenty-two cases of active HBV were reported in this subgroup (6.5% of patients), corresponding to 65 cases/1,000 patients. All six studies in high-burden countries reported cases of HBV. 3,024 patients received TNFα inhibitors in low-burden countries across 16 studies, with 11 cases of active HBV reported (0.4% of patients). Half of studies in low-burden countries reported no cases of HBV. Of the eight studies in the low-burden subgroup which reported HBV cases, all cases were reactivation of previous or chronic HBV infection, except for one case in which the patient was not tested for HBc or HBs antibodies prior to TNFα-inhibitor therapy (Ogata et al., 2016). There were 4 cases/1,000 patients in the low-burden subgroup. These data show a significant difference in the number of HBV cases/1,000 TNFα-inhibitor patients between countries with a high HBV burden and countries with a low HBV burden (*p* < .00001) (Figure 2).

### HCV reactivation risk with TNFα inhibitors

Six studies were identified which reported the number of cases of HCV reactivation in patients receiving TNFα inhibitors, of which four studies also reported HBV infections. The median number of study participants was 31 (range 6–67), with publication years ranging from 2010 to 2015. Extended study characteristics are outlined in the Supplementary Appendix Table SC3.

A total of 143 participants received therapy with at least one TNFα inhibitor, namely adalimumab, etanercept and/or infliximab. None of the six studies treated patients with golimumab or certolizumab pegol. Inflammatory conditions treated were RA, AS, PsA, CD, and PsO. One study did not report age or gender distribution for the TNFα-inhibitor exposed cohort, however in the remaining studies 52% of patients were female. Of the total patients exposed to TNFα inhibitors, three cases of HCV reactivation were reported (2.1% of patients), defined by an increase in HCV viral load. Two cases were reported in a single Spanish study (Navarro et al., 2013) and the remaining case was reported in an Italian study (Caporali et al., 2010). All cases of reactivation occurred in HCV positive patients. Overall, there were 21 HCV cases/1,000 patients.

Studies were subdivided according to continent. Five of the six studies were conducted in Europe, of which four were conducted in Italy. One study was conduction in Asia (Taiwan). Due to the limited number of studies and regional distribution, we were unable to perform subgroup statistical analysis between regions in the HCV group.

## Discussion

This is the first systematic review to focus on global regional differences in the risk of TB, HBV and HCV in patients treated with TNFα inhibitors, increasing the number of studies reviewed from 52 in a previous rheumatic disease-restricted review (Sartori et al., 2020) to 84 studies by placing no limitation on patient disease. Furthermore, observational, and post-marketing reports were included to focus the review on real-world outcomes.

This study found that the number of TB cases/1,000 TNFα-inhibitor patients was 4-fold higher in Asia, South America, and Africa (14/1,000) compared with Europe, North America, and Australasia (3/1,000). The findings expand upon a recent systematic review, which reported a higher frequency of TB among patients with rheumatic diseases receiving TNFα inhibitors in Asia (13/1,000) and South America (12/1,000) compared with Europe (6/1,000) and North America (4/1,000) (Sartori et al., 2020). The continental differences in TB frequency in TNFα-inhibitor patients observed in this systematic review also correlate with differences in TB burden in the general population (World Health Organization (WHO), 2021). The present study identified a particularly low TB burden in North America and Australasia, with no reported TB cases in TNFα-inhibitor patients in Australasia. Of those who developed TB in North America, several of the cases could be accounted for by the presence of additional risk factors, such as a history of TB exposure. One North American study found that TNFα-inhibitor patients who developed TB were less likely to be ethnically white or non-Hispanic and more likely to have co-morbidities such as diabetes or chronic renal disease (Winthrop et al., 2013). This highlights that in low-burden continents, TB risk in patients receiving TNFα inhibitors appears to be influenced by pre-existing risk factors.

A 3-fold higher frequency of TB was observed in patients receiving TNFα inhibitors in WHO-defined high-burden TB countries (19/1,000) compared with other regions (6/1,000). This may be explained by the fact that 86% of new TB cases in 2020 could be accounted for by the top 30 high-burden countries (World Health Organization (WHO), 2021), suggesting that TB risk in TNFα-inhibitor patients correlates with TB burden. The findings suggest that regional prevalence is a key factor determining risk of TB in TNFα-inhibitor patients. It is important to note, however, that some included studies performed in high-burden countries and continents reported no cases of TB in TNFα-inhibitor patients. This may be attributable to the use of prophylaxis. Previous studies have demonstrated that prophylaxis with standard anti-TB medication before or during TNFα-inhibitor therapy prevents TB reactivation (Carmona et al., 2005). However, due to incomplete reporting of these measures in the included studies, the present study was unable to assess the impact of prophylaxis on TB outcomes.

This was also the first systematic review to explore the interregional differences in HBV risk in TNFα-inhibitor patients. There was no significant difference in the frequency of HBV infection in TNFα-inhibitor patients in Asia compared with Europe. Given that continental differences in HBV burden are reported in the general population, with WHO South-East-Asian and Western-Pacific regions reporting a higher prevalence of chronic HBV in the general population compared with Europe (World Health Organization (WHO), 2017), this was an unexpected finding, but can possibly be explained by weak evidence supporting an association of HBV reactivation with TNFα inhibitors. Studies which have analyzed the association of TNFα inhibitors with HBV have yielded inconsistent results (Pérez-Alvarez et al., 2011), with existing systematic reviews reporting an overall low HBV reactivation rate in TNFα-inhibitor patients (Cantini et al., 2014; Moghoofei et al., 2018). This is reflective of the ambiguous role of TNFα in HBV viral clearance. TNFα is thought to play a role in the apoptosis of HBV-infected cells and in the inhibition of viral replication (Watashi et al., 2013). However, without contribution from other crucial mediators such as IFNƴ, IL1-β and cytotoxic CD8^+^ T lymphocytes (Yang et al., 2010), it is unlikely that TNFα alone can achieve these effects. The attributability of HBV reactivation to TNFα inhibition is therefore not certain.

When comparing the number HBV cases/1,000 TNFα-inhibitor patients according to general burden of chronic HBV by country, a >15-fold higher frequency of HBV was observed in TNFα-inhibitor patients treated in high-burden countries (65/1,000) compared with low-burden countries (4/1,000). This highlights the importance of considering regional differences in burden when determining HBV risk in TNFα-inhibitor patients. According to a 2018 report, general prevalence of chronic HBV infection varies between regions of Europe (European Association for the Study of the Liver (EASL), 2018). Whilst Western Europe has an estimated chronic HBV prevalence of <1%, estimated prevalence is far higher in Eastern Europe at <5%, with prevalence as high as 8% in Uzbekistan. Regional differences are similarly seen across Asia, where chronic HBV prevalence in Japan is estimated at <2%, compared to >8% in China (Yuen et al., 2018). HBV risk in TNFα-inhibitor patients thus appears to correlate with chronic HBV regional, rather than continental, differences in prevalence. This is also supported by the fact that most reports of HBV in the low-burden subgroup were cases of reactivation of previous or chronic HBV infection. Most of these patients were also treated with concomitant immunosuppressive therapies, including methotrexate and azathioprine. This observation is in keeping with a previous study, which reported an association of HBV reactivation with combined immunosuppressive therapy in IBD patients (Loras et al., 2010). The use of concomitant immunotherapy is therefore a possible factor influencing the risk of HBV reactivation in patients treated with TNFα inhibitors in low-prevalence areas.

There have been no systematic reviews which have investigated the interregional differences in risk of HCV with TNFα inhibitors. Due to the low number of study patients (143) identified and a lack of global distribution of these studies, it was not possible to perform subgroup analysis to determine regional differences in HCV infection in TNFα-inhibitor patients. However, all reports of HCV in the present study were cases of reactivation in patients with concomitant HCV infection, highlighting that underlying prevalence may influence HCV risk in TNFα-inhibitor patients.

There are several limitations to this study. Only five small studies with equal spread across the globe were conducted in children (1 United States, 2 Europe, 1 Turkey and 1 Taiwan with 17–73 patients each). Thus, age is also unlikely to be a significant confounding factor causing the observed regional differences in this study. Ethnicity, social and health risk factors, and length of follow-up were often not detailed and therefore it not possible to determine if they had any confounding effect on the infection risk. TNFα inhibitors are routinely added to steroids or immune suppressant drugs no matter which region of the world patients live. Whether immune suppressants add to the burden of TB and/or viral hepatitis over and above any effect of TNFα inhibitors, particularly in high prevalence countries, cannot be answered by this systematic review. However, the observation that patients living in countries with low burden have very low rates of TB and viral hepatitis is unlikely to be significantly confounded by immune suppressants, and the key take home messages of this paper therefore remain unchanged. There were few studies treating patients with golimumab or certolizumab pegol. There were a low number of studies in some regions increasing the risk of reporting bias. For example, only one study in the TB group was conducted in Australasia and all South American studies were performed in Brazil. This was particularly limiting in studies investigating cases of HCV in TNFα-inhibitor patients, and thus we were unable to determine interregional differences in the HCV group. There is a need for more studies investigating HCV risk with TNFα-inhibitor therapies across diverse regional populations.

Clinicians considering treating patients with TNFα inhibitors should benefit from the improved understanding of the regional differences in frequency of active TB and HBV in TNFα-inhibitor patients described in this systematic review, in the screening decision-making process. According to the UK National Screening Committee, for a screening program to be effective a disease being screened for should be relatively common, and screening should lead to improved health outcomes and be reasonable in cost (National Screening Committee and U.K., 2003). Whilst it may be beneficial to universally screen TNFα-inhibitor patients in high-burden regions where risk of TB or HBV are 3%–8%, in regions where TB and HBV are uncommon and only one patient will be picked up out of 2,500 patients treated with TNFα inhibitors, a universal screening program does not appear to meet these criteria. In this regard, it is worth remembering that the original 2001 report of 70 cases of TB (Keane et al., 2001), although seeming a lot, was only 0.03% of the total patients treated with TNFα biologics, or 1 TB patient detected for every 3,333 screened. Given that cases of TB and HBV in regions with a low burden of these infections in the present study were often accounted for by the presence of additional risk factors, such co-morbidities and concomitant treatment, a shift towards an individualized, risk-factor based screening approach should be considered as an alternative to universal screening in these areas.

In summary, TB and HBV risk in TNFα-inhibitor patients correlates with infection burden in the general population, highlighting regional TB and HBV burden as a risk factor for these infections in patients receiving TNFα inhibitors. An improved understanding of regional differences in infection risk should help to streamline screening of TNFα-inhibitor patients in low-burden areas. Studies providing details of co-morbidities, vaccination status, ethnicity, social and health risk factors, and foreign travel, are required to home in further on cofounding factors which influence infection risk with TNFα inhibitors.

## Data Availability

The original contributions presented in the study are included in the article/Supplementary Material, further inquiries can be directed to the corresponding author.
