# Supplementary material for: Regional risk of tuberculosis and viral hepatitis with tumor necrosis factor-alpha inhibitor treatment: A systematic review
Source: Front Pharmacol. 2023 Jan 20;14:1046306. doi: 10.3389/fphar.2023.1046306 (PMC9894886; doi:10.3389/fphar.2023.1046306)
Supplement: Supplementary file 1 [file DataSheet1.docx]

Supplementary Material

Regional risk of tuberculosis and viral hepatitis with tumor necrosis factor-alpha inhibitor treatment: A systematic review

Nina Jahnich^1^, Peter D. Arkwright^1*^

^1^ Lydia Becker Institute of Immunology & Inflammation, Manchester Incubator Building, University of Manchester, Manchester, United Kingdom

**APPENDIX A. SEARCH TERMS**

**Table A.1.** Key terms used in the systematic search of PubMed, Embase, MEDLINE and Web of Science databases.

| **Database** | **Search Terms** |
| --- | --- |
| **Embase and MEDLINE**  (searched via Ovid) | ((incidence or incidences or risk).af.) **AND** ((tuberculosis or Mycobacterium tuberculosis or TB or viral hepatitis or HBV or HCV or hepatitis B virus or hepatitis C virus).af.) **AND** ((infection or reactiv* or worse* or exacerbat*).af.) **AND** ((anti-TNF or infliximab or etanercept or adalimumab or golimumab or certolizumab).af.) **AND** ((North America or Europe or United Kingdom or USA or Australia or New Zealand or low-prevalence or Africa or South Africa or Asia or China or India or South America or endemic).af.) **NOT** (review).af. |
| **PubMed** | (incidence or incidences or risk) **AND** (tuberculosis or “Mycobacterium tuberculosis” or TB or “viral hepatitis” or HBV or HCV or “hepatitis B virus” or “hepatitis C virus”) **AND** (“anti-TNF” or infliximab or etanercept or adalimumab or golimumab or certolizumab) **AND** (infection or reactiv* or worse* or exacerbat*) **AND** (“North America” or Europe or “United Kingdom” or USA or Australia or “New Zealand” or “low-prevalence” or Africa or “South Africa” or Asia or China or India or “South America” or endemic) |
| **Web of Science** | (TS=(incidence or incidences or risk)) **AND** (TS=(tuberculosis or Mycobacterium tuberculosis or TB or viral hepatitis or HBV or HCV or hepatitis B virus or hepatitis C virus)) **AND** (TS=(infection or reactiv* or worse* or exacerbat*)) **AND** (TS=(anti-TNF or infliximab or etanercept or adalimumab or golimumab or certolizumab)) **AND** (TS=(North America or Europe or United Kingdom or USA or Australia or New Zealand or low- prevalence or Africa or South Africa or Asia or China or India or South America or endemic)) |

**APPENDIX B. RISK OF BIAS EVALUATION**

**Table B.1.** Risk of bias evaluation for the 105 included studies using the Newcastle-Ottawa Quality Assessment Scale for Cohort Studies**.** Each asterisk represents a star awarded to a study according to customizable categories, outlined below. A maximum score of nine can be awarded to a study. Studies with a score of <5 were considered to have greater potential for bias.

| **Study** | **Selection** | **Comparability** | **Outcome** | **Total Score** |
| --- | --- | --- | --- | --- |
| Acar et al. (57) | ** |  | *** | 5 |
| Aggarwal et al. (34) | *** |  | *** | 6 |
| Agarwal et al. (28) | ** | * | *** | 6 |
| Al-Sohaim et al. (29) | *** |  | *** | 6 |
| Alawneh et al. (58) | *** | * | ** | 6 |
| Arguder et al. (59) | **** |  | *** | 7 |
| Atteno et al. (31) | *** | ** | *** | 8 |
| Borekci et al. (60) | *** | * | *** | 7 |
| Bracaglia et al. (32) | * |  | *** | 4 |
| Byun et al. (61) | *** | ** | *** | 8 |
| Cagatay et al. (62) | *** |  | ** | 5 |
| Caporali et al. (50) | *** |  | ** | 5 |
| Carmona et al. (52) | ** | ** | *** | 7 |
| Chan et al. (63) | *** | * | *** | 7 |
| Charpin et al. (64) | *** |  | ** | 5 |
| Chiu et al. (65) | **** |  | *** | 7 |
| Chiu et al. (66) | ** | * | ** | 5 |
| Chiu et al. (67) | *** |  | ** | 5 |
| Cho et al. (40) | ** |  | *** | 5 |
| Chung et al. (68) | ** |  | *** | 5 |
| Ciang et al. (69) | *** | * | *** | 7 |
| Costa et al. (70) | *** |  | *** | 6 |
| Delabaye et al. (71) | *** | * | *** | 7 |
| Dixon et al. (72) | **** | * | *** | 8 |
| du Toit et al. (37) | *** | * | *** | 7 |
| Elbek et al. (73) | *** |  | *** | 6 |
| Ergun et al. (74) | *** | * | *** | 7 |
| Favalli et al. (75) | *** | ** | *** | 8 |
| Fidan et al. (76) | *** |  | *** | 6 |
| Fortes et al (77) | **** | ** | *** | 9 |
| Garcia-Vidal et al. (43) | *** |  | ** | 5 |
| Garziera et al. (78) | *** | * | * | 6 |
| Giardina et al. (79) | *** |  | ** | 5 |
| Gomes et al. (80) | *** | ** | *** | 8 |
| He et al. (81) | **** | * | *** | 8 |
| Hong et al. (82) | **** | ** | ** | 8 |
| Hsin et al. (83) | *** | * | *** | 7 |
| Jo et al. (84) | *** |  | *** | 6 |
| Jung et al. (85) | *** | * | ** | 6 |
| Jung et al. (86) | *** | * | ** | 6 |
| Kaptan et al. (87) | *** | ** | ** | 7 |
| Ke et al. (88) | **** | * | *** | 8 |
| Kim et al. (89) | **** | ** | ** | 8 |
| Kim et al. (90) | *** | ** | *** | 8 |
| Kim et al. (91) | **** | ** | *** | 9 |
| Kisacik et al. (92) | *** | ** | *** | 8 |
| Klein et al. (93) | *** | * | *** | 7 |
| Kwon et al. (94) | *** | ** | *** | 8 |
| Lan et al. (95) | *** | ** | *** | 8 |
| Lawrance et al. (39) | *** |  | ** | 5 |
| Lee et al. (96) | *** | ** | *** | 8 |
| Lee et al. (97) | *** | ** | *** | 8 |
| Lee et al. (98) | *** | ** | *** | 8 |
| Lee et al. (47) | *** | ** | ** | 7 |
| Liao et al. (99) | **** | ** | *** | 9 |
| Lim et al. (100) | **** | * | *** | 8 |
| Lim et al. (101) | **** | ** | ** | 8 |
| Lin et al. (102) | *** |  | *** | 6 |
| Mori et al. (103) | **** | * | ** | 7 |
| Morisco et al. (45) | *** | * | * | 5 |
| Nakamura et al. (104) | **** | * | *** | 8 |
| Namba et al. (41) | *** | ** | ** | 7 |
| Navarro et al. (105) | *** |  | *** | 6 |
| Navarro et al. (49) | *** | * | *** | 7 |
| Nisar et al. (106) | **** | * | ** | 7 |
| Nobre et al. (107) | *** | * | *** | 7 |
| O’Donnell et al. (108) | ** |  | *** | 5 |
| Ogata et al. (48) | *** | * | *** | 7 |
| Ohno et al. (109) | *** | * | *** | 7 |
| Pettipher et al. (38) | *** |  | *** | 6 |
| Prignano et al. (110) | *** |  | *** | 6 |
| Rahman et al. (111) | ** |  | *** | 5 |
| Rahman et al. (35) | ** |  | ** | 4 |
| Rahman et al. (36) | ** |  | *** | 5 |
| Rotar et al. (112) | *** | ** | *** | 8 |
| Rutherford et al. (113) | *** | * | ** | 6 |
| Ryu et al. (114) | *** | ** | *** | 8 |
| Samra et al. (115) | **** | ** | *** | 9 |
| Sanchez-Moya et al. (116) | *** | * | *** | 7 |
| Sayar et al. (42) | *** |  | *** | 6 |
| Seong et al. (117) | *** | ** | *** | 8 |
| Shen et al. (118) | *** | ** | *** | 8 |
| Shimabuco et al. (119) | *** | ** | *** | 8 |
| Sichletidis et al. (120) | *** | * | *** | 7 |
| Stoll et al. (121) | * |  | *** | 4 |
| Suwannalai et al. (122) | ** | * | *** | 6 |
| Takeuchi et al. (123) | ** |  | *** | 5 |
| Tarkiainen et al. (33) | ** |  | *** | 5 |
| Taxonera et al. (124) | **** | ** | *** | 9 |
| Ting et al. (125) | **** |  | *** | 7 |
| Tong et al. (126) | ** |  | ** | 4 |
| Torii et al. (30) | *** |  | *** | 6 |
| van der Have et al. (127) | *** | ** | *** | 8 |
| Vassilopoulos et al. (44) | *** | * | *** | 7 |
| Vuyyuru et al. (128) | *** |  | *** | 6 |
| Wang et al. (129) | *** | * | *** | 8 |
| Watanabe et al. (130) | *** | ** | * | 6 |
| Watanabe et al. (131) | **** | ** | ** | 8 |
| Winthrop et al. (51) | ** | * | *** | 6 |
| Wolfe et al. (132) | **** | * | ** | 7 |
| Ye et al. (133) | *** | ** | *** | 8 |
| Ye et al. (134) | *** | ** | *** | 8 |
| Yoo et al. (135) | *** | * | ** | 6 |
| Yoo et al. (136) | *** | * | *** | 7 |
| Zhou et al. (137) | ** |  | *** | 5 |

**Selection**

1. Representativeness of the exposed cohort

a) truly representative of the average PATIENT EXPOSED TO TNFα INHIBITORS in the community *

b) somewhat representative of the average PATIENT EXPOSED TO TNFα INHIBITORS in the community

c) selected group of users eg nurses, volunteers

d) no description of the derivation of the cohort

1. Selection of the non-exposed cohort

a) drawn from the same community as the exposed cohort *

b) drawn from a different source

c) no description of the derivation of the non-exposed cohort

1. Ascertainment of exposure

a) secure record (eg surgical records) *

b) structured interview *

c) written self-report

d) no description

1. Demonstration that outcome of interest was not present at start of study

a) yes *

b) no

**Comparability**

1. Comparability of cohorts on the basis of the design or analysis

a) study controls for PRE-TREATMENT SCREENING (select the most important factor) *

b) study controls for any additional factor AGE AND GENDER (This criterion could be modified to indicate specific control for a second important factor.) *

**Outcome**

1. Assessment of outcome

a) independent blind assessment *

b) record linkage *

c) self-report

d) no description

1. Was follow-up long enough for outcomes to occur

a) yes, SIX MONTHS (select an adequate follow up period for outcome of interest) *

b) no

1. Adequacy of follow up of cohorts

a) complete follow up - all subjects accounted for *

b) subjects lost to follow up unlikely to introduce bias - small number lost - > 75 % (select an adequate %) follow up, or description provided of those lost) *

c) follow up rate < 75% (select an adequate %) and no description of those lost

d) no statement

**APPENDIX C. EXTENDED STUDY CHARACTERISTICS**

**Table C.1.** Characteristics of the 84 included observational studies and post-marketing reports which report development of active TB in patients receiving TNFα inhibitors.

| **Study** | **Study Design** | **Year** | **Country** | **Continent** | **n exposed** | **n events active TB** | **Age (years)**  **Range or mean +/- SD** | **n female** | **Condition(s) treated** | **Biologic(s)** |
| --- | --- | --- | --- | --- | --- | --- | --- | --- | --- | --- |
| Acar et al. (57) | OS, RS, SC | 2017 | Turkey | Asia | 73 | 1 | 3-17 | 41 | UV, JIA, IBD, PsO, S, BD | ADA  ETA  IFX |
| Agarwal et al. (28) | OS, RS, SC | 2018 | India | Asia | 69 | 8 | *36 +/- 9,  35 +/- 16 | 34 | UC, CD | IFX |
| Aggarwal et al. (34) | OS, RS, SC | 2009 | USA | North America | 84 | 0 | 48 +/- 17 | 51 | RA, PsA, AS, JIA, V | ETA |
| Al-Sohaim et al. (29) | OS, RS, SC | 2021 | Saudi Arabia | Asia | 391 | 0 | 38 +/- 18 | 230 | RA, IBD, PsO, JIA, AS, CTD, HS, SI, UV, V | ADA |
| Alawneh et al. (58) | OS, RS, SC | 2014 | Jordan | Asia | 199 | 3 | 6-75 | 98 | RA, AS, IBD, JIA, BD, UV | ADA  ETA  IFX |
| Arguder et al. (59) | OS, PS, SC | 2020 | Turkey | Asia | 393 | 1 | NR | 225 | NR | ADA  ETA  IFX  GOL  CTZ |
| Atteno et al. (31) | OS, RS, SC | 2014 | Italy | Europe | 80 | 0 | *51 +/- 12,  51 +/- 12 | 56 | PsA | ADA ETA  IFX |
| Borekci et al. (60) | OS, RS, SC | 2015 | Turkey | Asia | 1964 | 16 | 39 +/- 14 | 955 | AS, RA, BD | ADA  ETA IFX |
| Bracaglia et al. (32) | OS, RS, SC | 2012 | Italy | Europe | 25 | 0 | 1 - 4 | 17 | JIA | ETA |
| Byun et al. (61) | OS, RS, MC | 2015 | South Korea | Asia | 873 | 25 | 5 - 81 | 294 | CD, UC | ADA  IFX |
| Cagatay et al. (62) | OS, PS, SC | 2010 | Turkey | Asia | 702 | 6 | 40 +/-14 | 379 | AS, RA, SpA, PsA, JRA, UC, BD | ADA  ETA  IFX |
| Carmona et al. (52) | OS, RS, MC | 2005 | Spain | Europe | 4102 | 34 | 50 +/- 15 | 2729 | RA, AS, PsA, JIA | ADA  ETA  IFX |
| Chan et al. (63) | OS, RS, SC | 2018 | Taiwan | Asia | 955 | 15 | 48 +/- 16 | 603 | RA, SpA | ADA  ETA  GOL |
| Chiu et al. (65) | OS, RS, SC | 2011 | Taiwan | Asia | 147 | 1 | 11-82 | 40 | PsO, PsA | ADA  ETA |
| Chiu et al. (66) | OS, RS, MC | 2017 | Taiwan | Asia | 9166 | 130 | *50 +/- 15,  51 +/- 16 | 6051 | RA, AS, PsO, PsA | ADA  ETA |
| Chiu et al. (67) | OS, RS, MC | 2014 | Taiwan | Asia | 2238 | 58 | 56 (mean) | 1392 | RA | ADA  ETA |
| Ciang et al. (69) | OS, RS, MC | 2020 | Hong Kong | Asia | 853 | 17 | NR | NR | SpA | ADA  ETA  IFX  GOL  CTZ |
| Delabaye et al. (71) | OS, PS, MC | 2010 | Belgium | Europe | 575 | 4 | 19-99 | 419 | RA | IFX |
| Dixon et al. (72) | OS, PS, MC | 2010 | United Kingdom | Europe | 10,712 | 40 | 56 +/- 12 | 8141 | RA | ADA  ETA  IFX |
| du Toit et al. (37) | OS, RS, MC | 2020 | South Africa | Africa | 174 | 20 | 18-46 | 73 | NR | ADA  ETA  IFX  GOL |
| Elbek et al. (73) | OS, PS, SC | 2009 | Turkey | Asia | 240 | 2 | 14-70 | 123 | AS, RA, PsA, SScl, JRA, ASD | ETA  IFX |
| Ergun et al. (74) | OS, PS, MC | 2015 | Turkey | Asia | 370 | 4 | 46 +/- 1 | 172 | PsO | ADA  ETA  IFX |
| Favalli et al. (75) | OS, RS, MC | 2009 | Italy | Europe | 1064 | 5 | 55 +/- 16 | 885 | RA | ADA  ETA IFX |
| Fortes et al. (77) | OS, RS, SC | 2020 | Brazil | South America | 61 | 4 | NR | NR | IBD | ADA  IFX |
| Garcia-Vidal et al. (43) | OS, RS, SC | 2009 | Spain | Europe | 94 | 4 | 15-85 | 64 | R, CD, AS, PsO, JRA | IFX |
| Garziera et al. (78) | OS, RS, SC | 2017 | Brazil | South America | 176 | 6 | 51 +/- 12 | 115 | RA, AS, PsA, JIA, SpA, JRA, BD, GWP | ADA  ETA  IFX  GOL |
| Gomes et al. (80) | OS, PS, MC | 2015 | Brazil | South America | 262 | 6 | * 54 +/- 12,  43 +/- 10, 56 +/- 9, 13 +/- 4 | 148 | RA, AS, JIA, PsA | ADA  IFX |
| He et al. (81) | OS, PS, SC | 2013 | China | Asia | 40 | 2 | NR | NR | RA, AS, PsA | ETA  IFX |
| Hong et al. (82) | OS, RS, MC | 2017 | South Korea | Asia | 3730 | 62 | NR | NR | UC, CD | ADA IFX |
| Hsin et al. (83) | OS, RS, MC | 2015 | Taiwan | Asia | 111 | 1 | 10 +/- 3 | 43 | JIA | NR |
| Jo et al. (84) | OS, RS, SC | 2013 | South Korea | Asia | 101 | 1 | 40 +/- 16 | 50 | CD, RA, AS, UC, PsO | ADA  ETA  IFX |
| Jung et al. (85) | OS, RS, MC | 2015 | South Korea | Asia | 8421 | 102 | 38 +/- 15 | 3469 | RA, AS, PsA, IBD | ADA  ETA  IFX |
| Jung et al. (86) | OS, PS, SC | 2015 | South Korea | Asia | 137 | 4 | 17 - 73 | 60 | RA, AS, IBD, SpA. PsA | ADA  ETA  IFX |
| Kaptan et al. (87) | OS, RS, SC | 2021 | Turkey | Asia | 389 | 7 | *46 +/- 0.5, 48 +/- 14 | 180 | AS, PsA, RA | ADA  ETA  IFX  CTZ |
| Ke et al. (88) | OS, RS, MC | 2013 | Taiwan | Asia | 829 | 9 | 52 +/- 13 | 619 | RD, SLE, PsO AS, CD, V, My, SScl, SS | ADA  ETA |
| Kim et al. (89) | OS, RS, SC | 2011 | South Korea | Asia | 354 | 3 | 35 +/- 11 | 56 | AS | ADA  ETA  IFX |
| Kim et al. (90) | OS, RS, MC | 2015 | South Korea | Asia | 376 | 16 | 32 +/- 13 | 121 | CD, UC | ADA  IFX |
| Kim et al. (91) | OS, RS, SC | 2014 | South Korea | Asia | 558 | 14 | *36 +/- 12, 51 +/- 13 | 256 | AS, RA | ADA  ETA  IFX  GOL  CTZ |
| Kisacik et al. (92) | OS, RS, MC | 2016 | Turkey | Asia | 7768 | 73 | *43 +/- 13, 43 +/- 14 | 4095 | RA, AS, PsA, BD | ADA  ETA  IFX |
| Klein et al. (93) | OS, PS, SC | 2013 | Czech Republic | Europe | 305 | 2 | 44 +/- 15 | 165 | RA, AS, PsA, JIA | ADA  ETA  IFX |
| Kwon et al. (94) | OS, RS, SC | 2014 | South Korea | Asia | 777 | 11 | 44 +/- 16 | 368 | RA, SA, IBD, ASD, BD, JIA, PsO | ADA  ETA  IFX |
| Lawrance et al. (39) | OS, RS, MC | 2010 | Australia, New Zealand | Australasia | 626 | 0 | 1 - 81 | 337 | CD, UC | ADA IFX |
| Lee et al. (96) | OS, RS, SC | 2013 | South Korea | Asia | 509 | 9 | 18- 85 | 268 | ASD, AS, BD, CD, GvHD, JRA, PsO, RA, UC | ADA  ETA  IFX |
| Lee et al. (97) | OS, RS, SC | 2021 | South Korea | Asia | 1434 | 21 | 31 +/- 14 | 494 | CD, UC | ADA  IFX  GOL |
| Lee et al. (98) | OS, PS, SC | 2018 | Hong Kong | Asia | 107 | 1 | NR | NR | CD, UC, IBD U, RA, AS, PsO, PsA | ADA  ETA  IFX  CTZ  GOL |
| Lio et al. (99) | OS, RS, MC | 2016 | Taiwan | Asia | 5255 | 188 | *49 +/- 13, 49.9 +/- 12.5 | 4372 | RA | ADA  ETA |
| Lim et al. (100) | OS, RA, MC | 2016 | Taiwan | Asia | 5349 | 80 | 55 +/- 13 | 4295 | RA | ADA  ETA |
| Lim et al. (101) | OS, RS, SC | 2017 | Taiwan | Asia | 835 | 24 | *52 +/- 15, 51 +/- 13, 48 +/- 13 | 680 | RA | ADA  ETA  GOL |
| Namba et al. (41) | PM, PS, MC | 2022 | Japan | Asia | 251 | 4 | NR | 153 | UV | ADA |
| Nisar et al. (106) | OS, RS, SC | 2015 | United Kingdom | Europe | 239 | 1 | NR | NR | RA, PSA, SpA, JIA | ADA  ETA  IFX GOL  CTZ |
| Nobre et al. (107) | OS, PS, SC | 2012 | Brazil | South America | 157 | 3 | 47 +/- 15 | 99 | RA, AS, PsA | IFX |
| O’Donnel et al. (108) | OS, RS, SC | 2011 | Ireland | Europe | 271 | 1 | 26 - 45 | 149 | CD, UC | IFX |
| Ogata et al. (48) | OS, PS, MC | 2016 | Japan | Asia | 1693 | 1 | 35 +/- 12 | 584 | CD | ADA |
| Ohno et al. (109) | PM, PS, MC | 2019 | Japan | Asia | 656 | 2 | 10 -78 | 154 | BD | IFX |
| Pettipher et al. (38) | OS, PS, MC | 2016 | South Africa | Africa | 86 | 0 | 29 - 77 | 68 | RA | ADA  ETA  IFX |
| Rahman et al. (111) | OS, RS, MC | 2020 | Canada | North America | 1577 | 1 | * 55 +/- 14, 57 +/- 13, 56 +/- 12 | 1298 | RA | IFX GOL |
| Rahman et al. (35) | OS, PS, MC | 2016 | Canada | North America | 303 | 0 | 45 +/- 12 | 114 | AS | IFX |
| Rahman et al. (36) | OS, PS, MC | 2020 | Canada | North America | 810 | 0 | * 45 +/- 12, 45 +/- 13 | 317 | AS | IFX GOL |
| Rotar et al. (112) | OS, RS, MC | 2020 | Slovenia | Europe | 2429 | 8 | 44 - 61 | NR | RA, AS, PsA | ADA  IFX  GOL  CTZ |
| Rutherford et al. (113) | OS, PS, MC | 2018 | United Kingdom | Europe | 16742 | 56 | 56 +/- 13 | 12724 | RA | ADA  ETA  IFX  CTZ |
| Samra et al. (115) | OS, PS, MC | 2015 | Saudi Arabia | Asia | 130 | 3 | 48 +/- 13 | 60 | RA | ADA  ETA  IFX |
| Sanchez-Moya et al. (116) | OS, PS, SC | 2011 | Spain | Europe | 144 | 1 | 18 - 82 | 86 | PsO | ADA  ETA  IFX |
| Seong et al. (117) | OS, RS, SC | 2007 | South Korea | Asia | 193 | 2 | *51 +/- 14, 51 +/- 12 | 165 | RA | ETA  IFX |
| Shen et al. (118) | OS, RS, SC | 2019 | China | Asia | 89 | 1 | 36 +/- 13 | 38 | BD | ADA  ETA IFX |
| Shimabuco et al. (119) | OS, RS, SC | 2020 | Brazil | South America | 218 | 11 | 49 +/- 13 | 69 | AS, PsA | ADA ETA  IFX  GOL  CTZ |
| Sichletidis et al. (120) | OS, RS, SC | 2006 | Greece | Europe | 613 | 11 | 22 - 71 | 416 | RA, AS, PSA, BD | ADA  ETA  IFX |
| Stoll et al. (121) | OS, RS, SC | 2017 | USA | North America | 1033 | 1 | 9 - 15 | 59 | JIA, IBD, V, UV, S, PsO, CTD, SLE, SjS, RFS, BD, HS, JDM, OM | ADA  ETA  IFX  GOL  CTZ |
| Suwannalai et al. (122) | OS, RS, SC | 2009 | Thailand | Asia | 100 | 1 | ≤50 - >70 | 68 | RA, SpA, uSpA, AS, PsA, RS, PsO, JIA, JD, CINCA, CD, IBD, BD | ETA  IFX |
| Takeuchi et al. (123) | PM, PS | 2008 | Japan | Asia | 5000 | 14 | 55 +/- 13 | 3950 | RA | IFX |
| Tarkiainen et al. (33) | OS, RS, MC | 2015 | Finland | Europe | 521 | 0 | 12 - 19 | NR | JIA | ADA  ETA  IFX |
| Taxonera et al. (124) | OS, PS, MC | 2018 | Spain | Europe | 192 | 1 | 43 +/- 14 | 85 | CD, UC | ADA  IFX  GOL |
| Ting et al. (125) | OS, PS, MC | 2021 | Taiwan | Asia | 635 | 1 | NR | NR | PsO | ADA  ETA |
| Tong et al. (126) | OS, PS, SC | 2015 | China | Asia | 172 | 6 | 39 +/- 16 | 27 | AS | ETA  IFX |
| Torii et al. (30) | PM, PS, MC | 2016 | Japan | Asia | 764 | 0 | 16 - 86 | 215 | PsO | IFX |
| van der Have et al. (127) | OS, RS, MC | 2014 | Netherlands | Europe | 611 | 3 | 23 - 42 | 396 | CD | ADA IFX |
| Vuyyuru et al. (128) | OS, RS, SC | 2021 | India | Asia | 59 | 3 | 30 +/- 16 | 18 | CD | IFX  ADA |
| Wang et al. (129) | OS, RS, MC | 2019 | Hong Kong | Asia | 2840 | 57 | NR | NR | RD, IBD, DD | ADA  ETA  IFX  GOL  CTZ |
| Watanabe et al. (130) | OS, RS, MC | 2016 | Japan | Asia | 7755 | 22 | *70 +/- 7, 60 +/- 13 | 6397 | RA | ADA |
| Winthrop et al. (51) | OS, RS, SC | 2013 | USA | North America | 8418 | 16 | 2 – 110 | 5387 | RA, CD, UC, AS, PsO | ADA  ETA  IFX |
| Wolfe et al. (132) | OS, PS,MC | 2004 | USA | North America | 6460 | 4 | 61 +/- 13 | 4750 | RA | ETA  IFX |
| Ye et al. (133) | OS, RS, MC | 2021 | China | Asia | 1968 | 21 | 21 - 35 | 579 | CD, UC | IFX |
| Yoo et al. (135) | OS, RS, MC | 2014 | South Korea | Asia | 175 | 3 | 18 - 86 | 61 | CD, UC, RA, AS | ADA  IFX |
| Yoo et al. (136) | OS, RS, SC | 2014 | South Korea | Asia | 1165 | 19 | NR | NR | IBD, RA, AS | ADA  ETA  IFX |
| Zhou et al. (137) | OS, RS, SC | 2015 | China | Asia | 70 | 2 | 28 +/- 1 | 21 | CD | IFX |

*Characteristics reported for different study subgroups. NR = not reported. OS = observational study, PM = post-marketing report, PS = prospective study, RS = retrospective study, SC = single centre, MC = multicentre. AS = ankylosing spondylitis, ASD = adult onset Still’s disease, BD = Behcet’s disease, CD = Crohn’s disease, CINCA = CINCA syndrome, CTD = connective tissue disease, DD = dermatologic disease, GvHD = graft vs host disease, GWP = granulomatosis with polyangiitis, HS = hidradenitis suppurativa, IBD = inflammatory bowel disease, JD = juvenile dermatomyositis, JIA = juvenile idiopathic arthritis, JRA = juvenile rheumatoid arthritis, My = myositis, OM = osteomyelitis, PsA = psoriatic arthritis, PsO = psoriasis, RA = rheumatoid arthritis, RD = rheumatic disease, RFS = recurrent fever syndrome, RS = Reiter’s syndrome, S = sarcoidosis, SI = sacroiliitis, SjS = Sjogren’s syndrome, SLE = systemic lupus erythematosus, SpA = spondylarthritis, SScl = systemic sclerosis, SS = sicca syndrome, UC = ulcerative colitis, uSpA = undifferentiated spondyloarthropathy, UV = uveitis, V = vasculitis. ADA = adalimumab, IFX = infliximab, ETA = etanercept, GOL = golimumab, CTZ = certolizumab pegol

**Table C.2.** Characteristics of the 23 included observational studies and post-marketing reports which report HBV reactivation or infection in patients receiving TNFα inhibitors.

| **Study** | **Study Design** | **Year** | **Country** | **Continent** | **n exposed** | **n events HBV** | **Age (years)**  **Range or mean±SD** | **n female** | **Condition(s) treated** | **Drug(s)** |
| --- | --- | --- | --- | --- | --- | --- | --- | --- | --- | --- |
| Caporali et al. (50) | OS, PS, MC | 2010 | Italy | Europe | 67 | 0 | 57 +/- 13 | 41 | RA, PsA, AS | ADA  ETA  IFX |
| Charpin et al. (64) | OS, PS, SC | 2009 | France | Europe | 21 | 0 | 57 +/- 3 | 13 | RA, PsA, AS | ADA  ETA  IFX |
| Cho et al. (40) | OS, RS, SC | 2012 | Taiwan | Asia | 7 | 3 | 38 - 48 | 1 | PsO | ADA  ETA |
| Chung et al. (68) | OS, RS, SC | 2009 | South Korea | Asia | 8 | 1 | 22 - 61 | 3 | RA, AS | ADA  ETA  IFX |
| Fidan et al. (76) | OS, RS, SC | 2021 | Turkey | Asia | 272 | 1 | 52 +/- 13 | 114 | SpA, RA, PsA, IBD, BD | ADA  ETA  IFX  GOL  CTZ |
| Garcia-Vidal et al. (43) | OS, RS, SC | 2009 | Spain | Europe | 94 | 1 | 15 - 85 | 64 | RA, CD, SpA, PsO | IFX |
| Giardina et al. (79) | OS, PS, SC | 2013 | Italy | Europe | 57 | 0 | 24 - 72 | 34 | RA, PsA, AS | ETA  IFX |
| Lan et al. (95) | OS, RS, SC | 2011 | Taiwan | Asia | 88 | 6 | 50 +/- 12 | 77 | RA | ADA  ETA |
| Lee et al. (47) | OS, RS, MC | 2022 | Korea, China, Taiwan, Japan | Asia | 191 | 14 | 20 - 70 | 55 | UC, CD | ADA  IFX  GOL |
| Mori et al. (103) | OS, PS, SC | 2011 | Japan | Asia | 161 | 1 | NR | NR | RA | ADA  ETA  IFX |
| Morisco et al. (45) | OS, RS, MC | 2013 | Italy | Europe | 7 | 2 | NR | NR | CD, UC | ADA  IFX |
| Nakamura et al. (104) | OS, RS, MC | 2016 | Japan | Asia | 48 | 1 | NR | NR | RA | ADA  ETA  IFX |
| Namba et al. (41) | PM, PS, MC | 2022 | Japan | Asia | 251 | 0 | NR | 153 | UV | ADA |
| Navarro et al. (105) | OS, RS | 2014 | Spain | Europe | 13 | 0 | 34 - 80 | 4 | PsO | ADA  ETA  IFX |
| Navarro et al. (49) | OS, RS, MC | 2013 | Spain | Europe | 4 | 0 | 31 - 57 | 2 | PsO | ETA  IFX |
| Ogata et al. (48) | OS, PS, MC | 2016 | Japan | Asia | 1693 | 1 | 35 +/- 12 | 584 | CD | ADA |
| Prignano et al. (110) | OS, RS, SC | 2011 | Italy | Europe | 17 | 0 | 36 - 74 | 4 | PsO | ADA  ETA |
| Ryu et al. (114) | OS, RS, MC | 2012 | South Korea | Asia | 49 | 3 | * 40 +/- 2, 47 +/- 16 | 19 | RA, AS | ADA  ETA  IFX |
| Sayar et al. (42) | OS, RS, SC | 2020 | Turkey | Asia | 90 | 0 | 46 +/- 13 | 38 | IBD, RA, AS, PsA | ADA  ETA  IFX  GOL  CTZ |
| Suwannalai et al. (122) | OS, RS, SC | 2009 | Thailand | Asia | 100 | 1 | ≤50 - >70 | 68 | RA, SpA, uSpA, AS, PsA, RS, PsO, JIA, JD, CINCA, CD, IBD, BD | ETA  IFX |
| Vassilopoulos et al. (44) | OS, PS, SC | 2010 | Greece | Europe | 131 | 1 | 52 +/- 16 | 81 | RA, AS, PsA, CD, U | ADA  ETA  IFX |
| Watanabe et al. (131) | OS, RS, SC | 2019 | Japan | Asia | 98 | 3 | NR | NR | RA | ADA  ETA  IFX  GOL  CTZ |
| Ye et al. (134) | OS, PS, MC | 2014 | China | Asia | 87 | 8 | *46 +/- 6,  16 - 59 | 48 | AS, RA, PsA | ETA  IFX |

*Characteristics reported for different study subgroups. NR = not reported. OS = observational study, PM = post-marketing report, PS = prospective study, RS = retrospective study, SC = single centre, MC = multicentre. AS = ankylosing spondylitis, BD = Behcet’s disease, CINCA = CINCA syndrome, CD = Crohn’s disease, IBD = inflammatory bowel disease, JIA = juvenile idiopathic arthritis, JD = juvenile dermatomyositis, PsA = psoriatic arthritis, PsO = psoriasis, RA = rheumatoid arthritis, RS = Reiter’s syndrome, SpA = spondylarthritis, UC = ulcerative colitis, uSpA = undifferentiated spondyloarthropathy, UV = uveitis, U = undifferentiated. ADA = adalimumab, IFX = infliximab, ETA = etanercept, GOL = golimumab, CTZ = certolizumab pegol

**Table C.3.** Characteristics of the six included observational studies which report HCV reactivation or infection in patients receiving TNFα inhibitors.

| **Study** | **Study Design** | **Year** | **Country** | **Continent** | **n exposed** | **n events HCV** | **Age (years)**  **Range or mean±SD** | **n female** | **Condition(s) treated** | **Drug(s)** |
| --- | --- | --- | --- | --- | --- | --- | --- | --- | --- | --- |
| Caporali et al. (50) | PS, MC | 2010 | Italy | Europe | 67 | 1 | 57 +/- 13 | 41 | RA, PsA, AS | ADA  ETA  IFX |
| Costa et al. (70) | RS, MC | 2014 | Italy | Europe | 15 | 0 | 50 - 71 | 9 | PsA | ADA  ETA |
| Lin et al. (102) | RS, SC | 2015 | Taiwan | Asia | 20 | 0 | 63 +/- 9 | 17 | RA | ADA  ETA |
| Morisco et al. (45) | RS, MC | 2013 | Italy | Europe | 6 | 0 | NR | NR | CD | ADA  IFX |
| Navarro et al. (49) | RS, MC | 2013 | Spain | Europe | 18 | 2 | 21 - 80 | 1 | PsO | ADA  IFX |
| Prignano et al. (110) | RS, SC | 2011 | Italy | Europe | 17 | 0 | 36 - 74 | 4 | PsO | ADA  ETA |

NR = not reported. OS = observational study, PM = post-marketing report, PS = prospective study, RS = retrospective study, SC = single centre, MC = multicentre. AS = ankylosing spondylitis, CD = Crohn’s disease, PsA = psoriatic arthritis, PsO = psoriasis, RA = rheumatoid arthritis. ADA = adalimumab, IFX = infliximab, ETA = etanercept.
